# Vanillic Acid Reduces Pain-Related Behavior in Knee Osteoarthritis Rats Through the Inhibition of NLRP3 Inflammasome-Related Synovitis

**DOI:** 10.3389/fphar.2020.599022

**Published:** 2021-02-15

**Authors:** Zhenyuan Ma, Zhengquan Huang, Li Zhang, Xiaochen Li, Bo Xu, Yancheng Xiao, Xiaoqing Shi, Haosheng Zhang, Taiyang Liao, Peimin Wang

**Affiliations:** ^1^ Department of Orthopedics, Affiliated Hospital of Nanjing University of Chinese Medicine, Nanjing, China; ^2^ Jiangsu Province Hospital of Chinese Medicine, Nanjing, China; ^3^ Key Laboratory for Metabolic Diseases in Chinese Medicine, First College of Clinical Medicine, Nanjing University of Chinese Medicine, Nanjing, China

**Keywords:** vanillic acid, knee osteoarthritis, synovial inflammation, NLRP3 inflammasome, pain 3

## Abstract

**Objectives**: Synovitis plays an important role in knee osteoarthritis (KOA) pain. The activation of the NOD-like receptor protein 3 (NLRP3) inflammasome in fibroblast-like synoviocytes (FLSs) promotes KOA development. In this study, we aimed to investigate whether vanillic acid (VA), a monomer derived from Chinese herbal medicines, could target NLRP3 inflammasome-related synovitis to reduce pain.

**Methods**: Rats in the KOA and KOA + VA groups were injected with monosodium iodoacetate (MIA) in the knee to induce KOA. From day 14, the KOA + VA group was given VA at 30 mg/kg every day via gastric intubation. FLSs were collected from the synovial tissues. We examined both the protein and gene expression of caspase-1, apoptosis-associated speck-like protein with a caspase recruitment domain (ASC), NLRP3, components of the NLRP3 inflammasome, and interleukin-1β (IL-1β) and IL-18 *in vivo* and *in vitro*.

**Results**: The upregulation of caspase-1, ASC, and NLRP3 in the KOA model were reduced by VA. VA also lowered the level of IL-1β and IL-18 in the KOA model. In addition, VA relieved pain-related behavior of KOA model rats and downregulated the pain mediators CGRP, NGF, and TrkA in FLSs. Interestingly, we also observed reduced synovial fibrosis in the animal experiments.

**Conclusion**: Our research showed that VA reduces synovitis and pain-related behaviors in a rat model of KOA, which provides the basis for further investigations into the potential therapeutic impact of VA in KOA.

## Introduction

Knee osteoarthritis (KOA) is one of the most common chronic degenerative bone diseases and joint pain is the main clinical symptom that needs to be solved urgently (Litwic et al., 2013). The occurrence and development of KOA are associated with all the tissues that make up the knee joint, such as the synovium, cartilage, subchondral bone, and subcondylar fat pads (Muratovic et al., 2019). KOA also leads to the alteration of the whole joint structure (Akhbari et al., 2020). Symptoms in KOA are also somewhat related to structure. As an immune-related disease, the inflammation of the synovium in KOA plays a vital role and leads directly to the related pain (Atukorala et al., 2013). Recent studies have revealed that the activation of the NOD-like receptor protein 3 (NLRP3) inflammasome is the beginning of inflammatory cascade amplification and is strongly correlated to hepatitis, pneumonia, nephritis, and other types of chronic aseptic inflammation (Ferrucci and Fabbri, 2018). NLRP3 is a molecular platform activated by caspase-1, which interacts with apoptosis-associated speck-like protein with a caspase recruitment domain (ASC) and procaspase-1, to assemble the NLRP3 inflammasome. Subsequently, the activated NLRP3 inflammasome cleaves procaspase-1, which leads to the maturation and secretion of interleukin- (IL-) 1β and IL-18 (Martinon et al., 2002). Our previous study showed that activation in KOA fibroblast-like synoviocytes (FLSs) promoted the development of synovitis (Zhao et al., 2018; Zhang et al., 2019).

Among many pain-related factors, the nerve growth factor (NGF) is a key regulator of KOA pain. High expression of NGF in the synovial fluid and cartilage of KOA patients has been widely reported. NGF antibodies have shown potential in the treatment of KOA pain. A similar situation occurs in tropomyosin receptor kinase A (TrkA), a high-affinity NGF receptor (Pecchi et al., 2014). Meanwhile, pain is associated with calcitonin gene-related peptide (CGRP), which is expressed in the sensory neurons that dominate the synovium. Studies have shown that CGRP levels from the KOA synovium are significantly increased and are highly correlated with KOA peripheral sensitization (Arnalich et al., 1994). In summary, NGF, TrkA, and CGRP are associated with pain, and changes in their expression can be used as biomarkers in KOA pain studies.

Chinese herbal medicine used in the clinical treatment of KOA has a long history. “Sanse powder,” which is created from eleven substances found in Chinese materia medica (CMMs) (Wu et al., 2020), *Forsythia suspensa*, *Glycyrrhiza uralensis*, *Salvia miltiorrhiza*, *Gentiana macrophylla*, *Chaenomeles sinensis*, *Strychnos nux-vomica*, *Ligusticum striatum* Hort, *Curcuma longa*, *Paeonia lactiflora*, *Notopterygium* root, and *Saposhnikovia divaricata*, is a classic prescription widely used, not only to relieve synovitis but also to effectively relieve KOA pain. We performed a high-performance liquid chromatography analysis on "Sanse powder" to determine the effective components, of which vanillic acid (VA) is one (Wu et al., 2020). VA is a well-known flavonoid that is rich in nuts, fruits, and herbs. In recent years, VA has been mentioned in some studies as having anti-inflammatory and analgesic effects (Wu et al., 2020). However, the mechanisms by which VA affects the inflammation of KOA and its effects on pain are still poorly understood. Whether VA can affect the NLRP3 inflammasome has not been described in detail. Therefore, in this study, we observed the intervention effects of VA on NLRP3 inflammasome activation, synovitis, and KOA pain-related behaviors/mediator *in vivo* or *in vitro*.

## Material and Methods

### Reagents

Vanillic acid was purchased from Yuanye Bio-Technology Co., Ltd. (Shanghai, China). The primers were supplied by Sangon Biotech (Shanghai, China). The enzyme-linked immunosorbent assay (ELISA) kits for IL-1β and IL-18 were supplied by Invitrogen (Life Technologies Corp. California, United States). Fetal bovine serum (FBS), bovine serum albumin (BSA), Dulbecco’s Modified Eagle’s Medium (DMEM), TRIzol, and 0.25% trypsin-ethylenediaminetetraacetic acid (trypsin-EDTA) were purchased from Gibco (Life Technologies Corp., California, United States). Cell Counting Kit-8 (CCK-8) was purchased from Dojindo (Kumamoto, Japan). Antibodies against NLRP3, ASC, caspase-1, CGRP, NGF, TrkA, and type I collagen were purchased from Abcam (Cambridge, United Kingdom). Monoidoacetate acid, type I collagenase, and dimethylsulfoxide (DMSO) were all obtained from Sigma (St Louis, USA). All other chemicals were of reagent grade. Goat anti-rabbit IgG H&L (HRP) and Picro Sirius Red Stain kit were also supplied by Abcam (Cambridge, United Kingdom).

### Rat KOA Model and Experimental Design

Twenty-four 3-month-old male Sprague-Dawley rats, weighing 280–320 g (provided by Beijing Vital River Laboratory Animal Technology Co., Ltd.), were fed in specific pathogen-free animal facilities under standard conditions (temperature 21 ± 1 °C, 50–80% relative humidity) with a 12:12 h light-dark cycle. Animal management was done in accordance with the Animal Care and Use Protocol approved by the Animal Care and Use Committee of our institution (201810A001). All animals were experimented on according to the National Institute of Health Animal Care and Use Guidelines. Rats were randomly divided into three groups: control (n = 8), KOA (n = 8), and KOA + VA (n = 8). The KOA and KOA + VA rats were injected with monosodium iodoacetate (MIA) in the knee to induced KOA (Rannou et al., 2016). Our previous data showed that the knee joint diameter was significantly larger than the control at the 14th day (Li et al., 2020). Therefore, from day 14, the KOA + VA group was given VA at 30 mg/kg (dissolved in 0.9% saline) every day via gastric intubation. On day 56, all rats were sacrificed by anesthesia and blood was collected. Knee joint tissues were collected for further experiments. We chose the VA dose based on a previous study published by another team (Huang et al., 2019). Animal number was estimated using PASS. When *α* = 0.05, the power = 0.9, the minimum detected difference is 1, and the standard deviation is 0.5, so the number should be ≥6 when the power reaches 0.9. Therefore, eight rats in each group were used. The animal experiments complied with the relevant regulations for animal experiments of the Ethics Committee at our institution.

### Paw Withdrawal Experiments on a Cold Plate

Before scarification, the rats were placed on a cold glass surface to determine their paw withdrawal time. The rats were briefly placed on a temperature-adjustable cold plate (0 ± 1 °C, 35,150,001, Ugo Basil SLR, Italy) and covered with an organic cylinder. We recorded the time between the start and the limb moving off the glass plate.

### Sirius Red Staining and Immunohistochemistry

The synovial tissues were fixed with 4% paraformaldehyde, embedded in paraffin, and sectioned; then, conventional Sirius red staining was used to observe tissue changes under a light microscope. Synovial tissues were paraffin-sectioned via immunohistochemical staining. The content of collagen in each group was also detected by immunohistochemical staining.

### Hematoxylin and Eosin Staining

Synovial tissues were fixed in 4% paraformaldehyde, then embedded in paraffin, and cut into thin slices for routine hematoxylin and eosin (HE) staining.

### Cell Culture

Primary FLSs were isolated from rat KOA. Briefly, synovial tissues were washed 2–3 times with cold phosphate-buffered saline (PBS), minced into pieces of 2–3 mm^2^, and then digested in 0.1% collagenase type II (Sigma, St. Louis, MO, USA) for 30 min. The solution was filtered through a cell strainer. After dissociation, fibroblasts were collected via centrifugation at 1,500 rpm for 4 min and cultured in DMEM supplemented with 10% fetal calf serum (FCS; Gibco, Thermo Fisher Scientific, Waltham, MA, USA) and antibiotics (100 U/ml penicillin, 100 μg/ml streptomycin; Invitrogen, CA, USA). Cells were identified as described in our previous studies (Li et al., 2020). Passages three to six of the synovial fibroblasts were used for the experiments. In the cell experiment, we used lipopolysaccharide (LPS) (5 μg/ml) to interfere with the synovial cells for 24 h to mimic a KOA environment and then added VA (5 μg/ml) to the VA + LPS group to intervene with the cells.

### Cell Cytotoxicity Assay

A CCK-8 kit was used to detect the cytotoxicity of VA to FLSs. FLSs were cultured in 96-well plates. When the cell density reached 85–90%, they were treated with different VA concentrations (0, 5, 10, and 20 μg/ml) for 24 or 48 h. Then, we added 10 μL of the CCK-8 solution to each well and placed the cells in the incubator for 3 h. The optical density of the wells was detected using a 450 nm microplate spectrophotometer. All experiments were repeated three times.

### Western Blotting Assay

Synovial tissues and FLSs were mixed with radioimmunoprecipitation assay (RIPA) lysate and grinded for 10–15 min. The samples were agitated on ice for 30 min and the supernatant was collected. The protein levels were quantified with a bicinchoninic acid (BCA) protein assay kit (Roche, Basel, Switzerland). Then, the protein samples were electrophoresed in sodium dodecylsulphate polyacrylamide gel electrophoresis (SDS-PAGE) to separate the protein bands. Proteins were transferred from gel onto a polyvinylidene fluoride (PVDF) membrane and blocked with 5% nonfat milk for 2 h. The membrane was incubated with the primary antibody (1: 1000) at 4 °C overnight and then with the second antibody for 2 h. Bands were visualized via exposure to the electrochemiluminescence (ECL) method, and the overall gray value of protein bands (average gray value area) was quantified. Glyceraldehyde-3-phosphate dehydrogenase (GAPDH) was used as the internal marker. The relative protein expression was taken as the target protein gray value/internal reference gray value.

### Real-Time Quantitative Polymerase Chain Reaction

RNA was isolated from synovial tissue and FLSs with Trizol (Invitrogen, CA, United States). Reverse transcription was performed using a first-strand cDNA synthesis kit (Takara, Otsu, Japan) according to the manufacturer’s instructions. Quantitative polymerase chain reaction (qPCR) was performed using Premix Ex Taq SYBR-Green PCR (Takara) according to the manufacturer’s instructions on an ABI PRISM 7300 device (Applied Biosystems, Foster City, CA, USA).

The primer was designed and synthesized by the Shanghai Biotechnology Service Company. Primers Sequences were as follows. Caspase-1: forward, 5′-ATGGCCGACAAGGTCCTGAGG-3′and reverse, 5′-GTG​ACA​TGA​TCG​CAC​AGG​TCT​CG-3′; NLRP3: forward, 5′-GAGCTGGACCTCAGTGACAATGC-3′and reverse, 5′-ACC​AAT​GCG​AGA​TCC​TGA​CAA​CAC-3′; IL-18: forward, 5′-TCT​GTA​GCT​CCA​TGC​TTT​CCG-3′ and reverse, 5′-GAT​CCT​GGA​GGT​TGC​AGA​AGA-3′; and IL-1β: forward, 5′-ACA​GCA​GCA​TCT​CGA​CAA​GAG​C-3′ and reverse, 5′-CCA​CGG​GCA​AGA​CAT​AGG​TAG​C-3′; CGRP: forward, 5′-ATC​TGG​TCC​TTC​CTC​ACA​CTG​TCC-3′ and reverse, 5′-TCA​TCC​GTC​TTC​AGC​TTG​GCA​TTC-3′; TrkA: forward, 5′-AGG​TTG​AAG​CCA​TTC​TCC​TG-3′ and reverse, 5′-TCT​CGG​TGG​TGA​ACT​TAC​GG-3′; NGF: forward, 5′-CCA​GCC​TCC​ACC​CAC​CTC​TTC-3′ and reverse, 5′-GCT​TGC​TCC​TGT​GAG​TCC​TGT​TG-3′; GAPDH: forward, 5′-TTC​ACC​ACC​ATG​GAG​AAG​GC-3′ and reverse, 5′-CTC​GTG​GTT​CAC​ACC​CAT​CA-3′; ASC: forward, 5′-AGA​GTC​TGG​AGC​TGT​GGC​TAC​TG-3′ and reverse, 5′-ATG​AGT​GCT​TGC​CTG​TGT​TGG​TC-3′. The PCR reactions (per well: 0.4 µL of forward and reverse primers, 10 µL 2 × ChamQ SYBR qPCR Master Mix (Low ROX Premixed), 1 µL CDNA, and 8.2 µL ddH_2_O; three replicate wells) were performed using an ABI 7500 qRT-PCR system (Applied Biosystems, United States). The following reaction conditions were employed: the first stage (predenaturation), 95 °C for 30 s; the second stage (denaturation), 95 °C for 10 s and 60 °C for 30 s; the third stage (melting curve), 95 °C for 15 s, 60 °C for 60 s, and 95 °C for 15 s. The relative expression of mRNA was adjusted using GAPDH as the internal reference and calculated using the method of 2^−ΔΔCt^.

### Enzyme-Linked Immunosorbent Assay (ELISA)

IL-1β and IL-18 levels in the culture media were determined using a commercially available rat IL-1β and IL-18 ELISA kit according to the manufacturer’s instructions. The rat peripheral serum and cell culture supernatants were collected and centrifuged at 10,000 rpm for 20 min at 4 °C.

### Statistical Analysis

The statistical analysis was performed using the SPSS 20.0 software (SPSS Inc., Chicago, IL, USA). Data are presented as the mean ± standard deviation. Group comparisons were assessed with one-way ANOVA or two-way ANOVA with Bonferroni’s *post h*oc test for comparison of multiple columns. A value of *p* < 0.05 (two-tailed) was considered statistically significant.

## Results

### VA Inhibits NLRP3 Inflammasome Activation *In Vivo*

**FIGURE 1:**
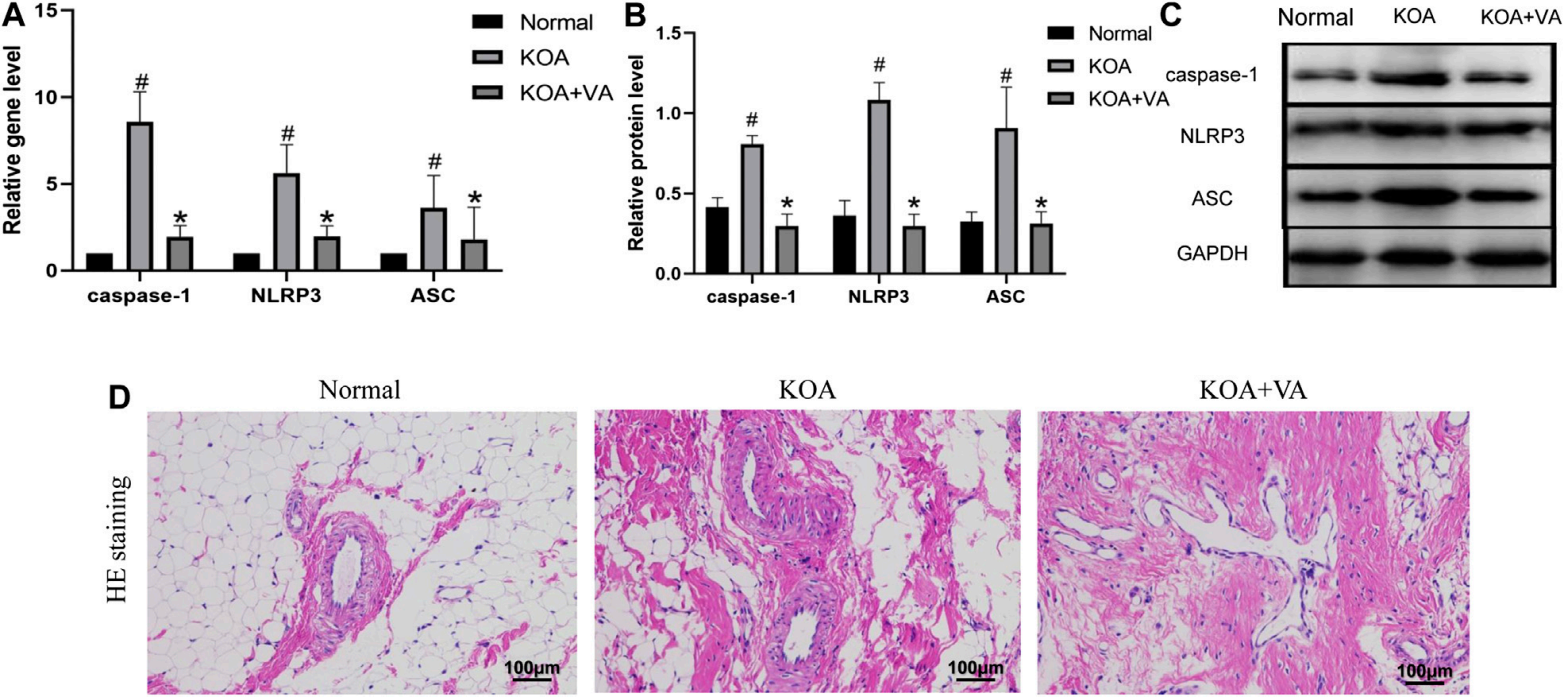
Vanillic acid (VA) inhibits NLRP3 inflammasome activation. **(A)** Relative gene expression of caspase-1, NLRP3, and ASC in synovial tissue of rats in each group. Compared with the normal group, *p* ^#^ < 0.05; compared with KOA + VA group, p* < 0.05. **(B and C)** Western blot analysis of the effects of VA on caspase-1, NLRP3, and ASC in rat synovial tissue. Compared with the normal group, *p* ^#^ < 0.05; compared with KOA + VA group, p* < 0.05. **(D)** Synovial tissues of each group stained with HE, 400 ×, scale bar = 100 μm.

To explore whether VA inhibits NLRP3 activation *in vivo*, we analyzed the expression of caspase-1, NLRP3, and ASC in the Normal group, KOA group, and KOA + VA group. The mRNA and protein expressions of caspase-1, NLRP3, and ASC (Figures 1A–C) in the KOA group were higher than those in the normal group (*p* < 0.05), whereas the KOA + VA group showed a reduction compared to the KOA group (*p* < 0.05). Compared with the KOA group, HE staining (Figure 1D) of rats treated with VA resulted in less inflammation infiltration and cell proliferation.

### VA Reduces Pain-Related Behavior/Mediator in Knee Osteoarthritis *In Vivo*

**FIGURE 2:**
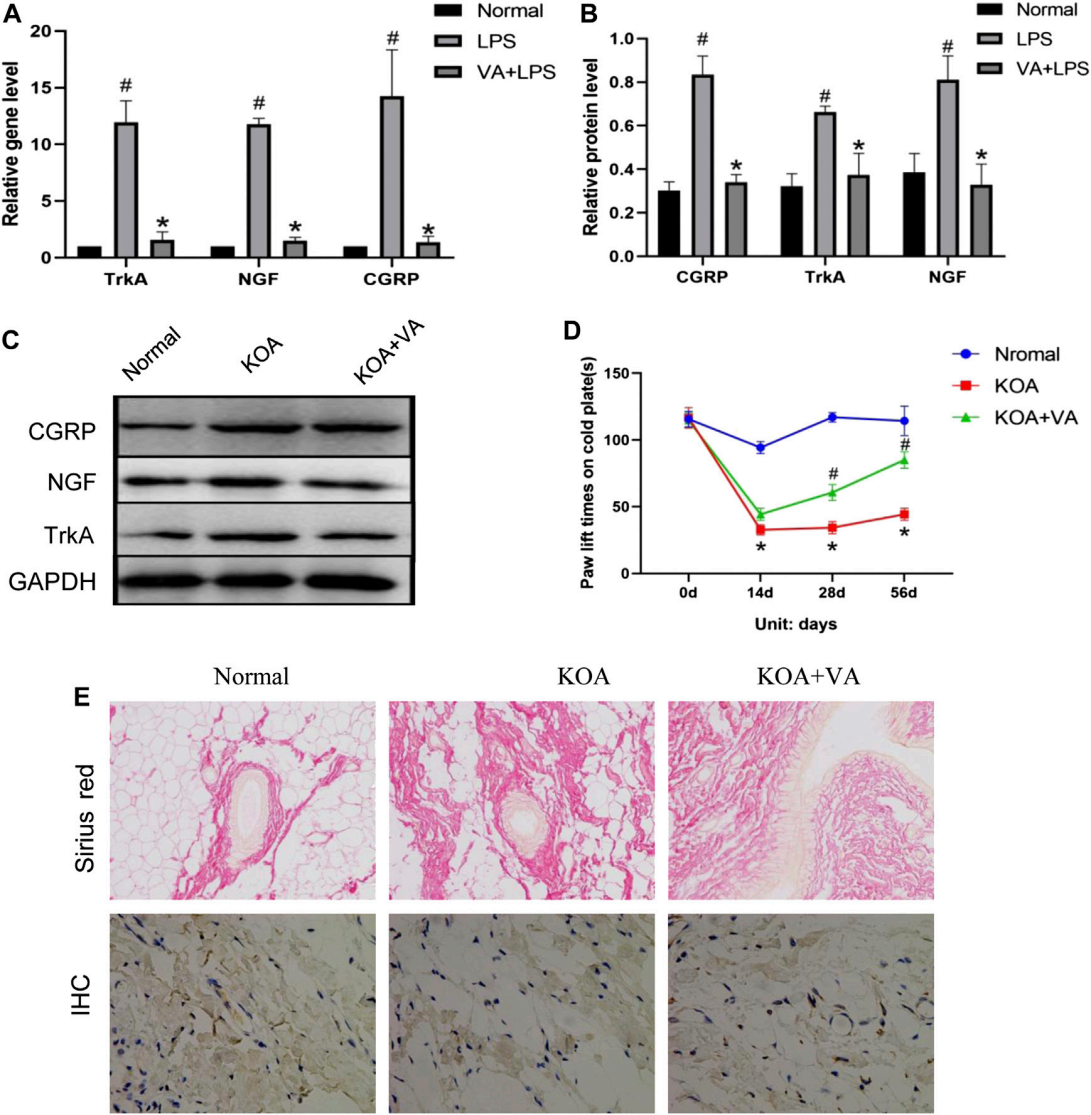
Vanillic acid (VA) relieves pain in knee osteoarthritis. **(A)** Relative gene expression of NGF, TrkA, and CGRP in synovial tissue of rats in each group. Compared with the normal group, *p* ^#^ < 0.05; compared with KOA + VA group, p* < 0.05. **(B and C)** Western blot analysis of the effects of VA on NGF, TrkA, and CGRP in rat synovial tissue. Compared with the normal group, *p* ^#^ < 0.05; compared with KOA + VA group, p* < 0.05. **(D)** Statistics of paw withdrawal time on a cold plate in each group. **(E)** Sirius red staining of synovium tissue, 200x, scale bar = 100 µm. Immunohistochemical detection of type I collagen in synovium tissue, 400×, scale bar = 100 µm.

To assess the effect of VA on pain-related behavior/mediator during KOA *in vivo*, we analyzed pain-related factors in the KOA and KOA + VA groups. The protein and mRNA expressions of NGF, TrkA, and CGRP in the synovial tissue of rats (Figures 2A–C) in the KOA + VA group were lower than those in the KOA group (*p* < 0.05). In the cold-plate paw withdrawal experiment (Figure 2D), compared with the blank group, the paw withdrawal time of the KOA group was significantly shortened (*p* < 0.05). In the KOA + VA group, the claw lift time was close to that of the blank group.

Interestingly, we observed the effects of VA on synovial fibrosis. In the type I collagen immunohistochemical analysis, Sirius red staining showed that the KOA group experienced a significant increase in collagen deposition, while the KOA + VA group showed a relative decrease (Figure 2E).

### VA Reduces Expression of IL-18 and IL-1β *In Vivo*

**FIGURE 3:**
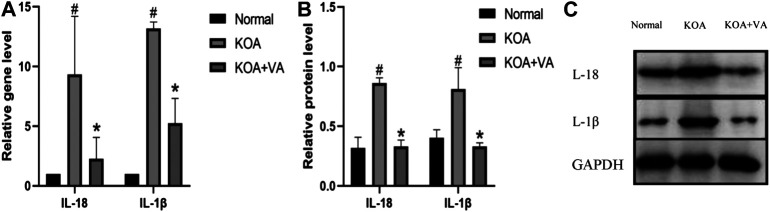
Vanillic acid (VA) reduces the expression of IL-18 and IL-1β. **(A)** Relative gene expression of IL-18 and IL-1β in synovial tissue of rats in each group. Compared with the normal group, *p* ^#^ < 0.05; compared with KOA + VA group, p* < 0.05. **(B and C)** Western blot analysis of the effects of VA on synovial tissue IL-18 and IL-1β in rats.

To evaluate the effect of VA on NLRP3 inflammasome activation, we further analyzed the gene expression of IL-18 and IL-1β via real-time quantitative PCR (Figure 3). The expressions of IL-18 and IL-1β under VA intervention were significantly lower than those without VA intervention (*p* < 0.05). The protein expression of these NLRP3 downstream proinflammatory factors showed the same trend.

### Effects of VA on FLSs Viability

**FIGURE 4:**
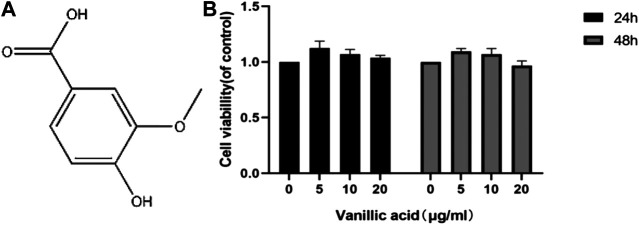
Effects of vanillic acid (VA) on fibroblast-like synoviocytes (FLSs) viability. **(A)** Chemical structure of VA. **(B)** The cells were cultured with increasing concentrations of vanillic acid (0, 5, 10, and 20 μg/ml) for 24 h and 48 h. Cell viability was measured using the CCK-8 kit.

We also used CCK-8 to determine the activity of synovial cells under the treatment of different concentrations of VA *in vitro* (Figure 4). The results showed that the activity of synovial cells was better at a dose of 5 μg/ml, although no statistical difference was observed. We decided to use VA at the minimal concentration of 5 μg/ml for subsequent experiments.

### VA Inhibits the Inflammatory Changes in FLSs

**FIGURE 5:**
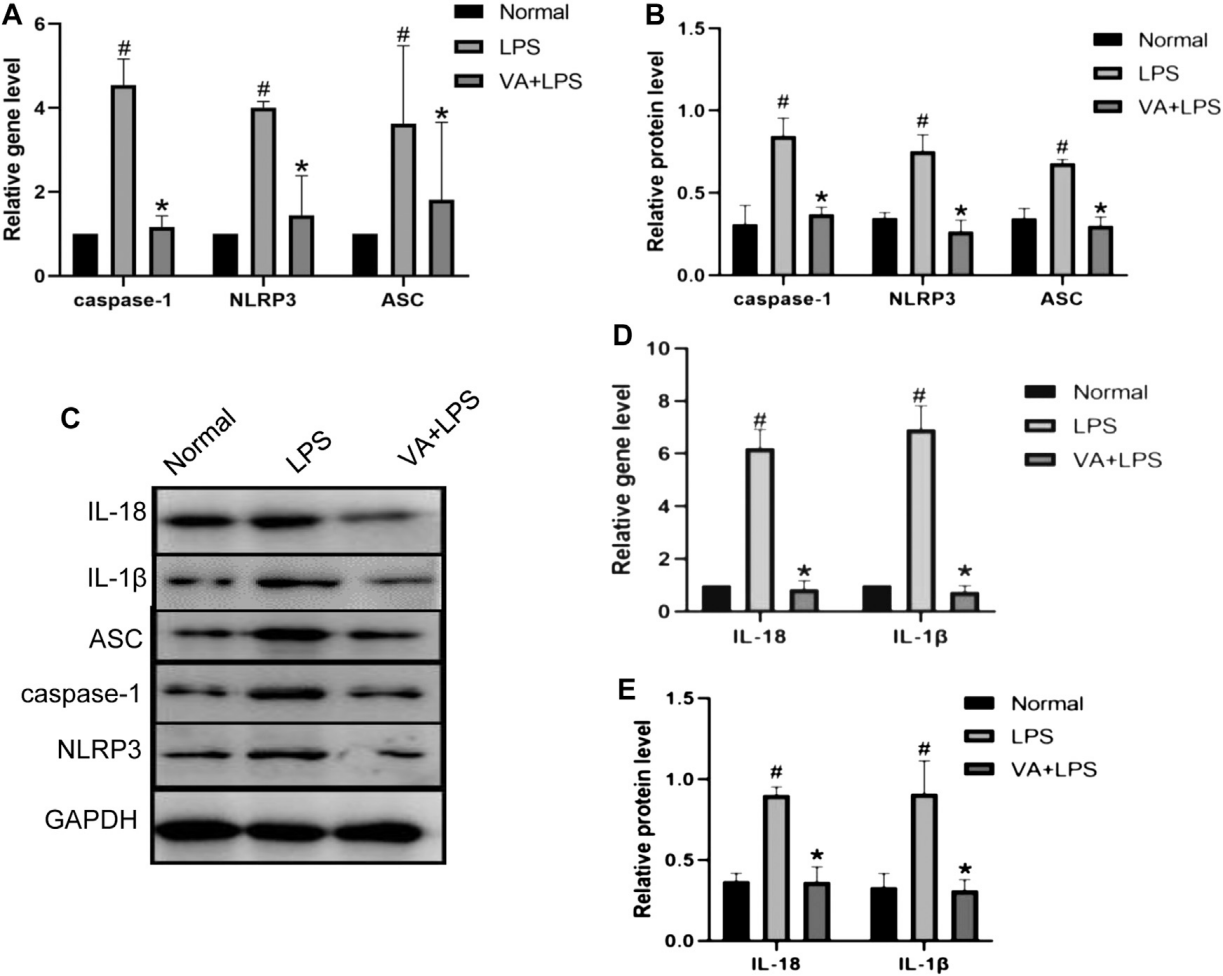
Vanillic acid (VA) inhibits the inflammatory changes in fibroblast-like synoviocytes (FLSs). **(A)** Relative gene expression of caspase-1, NLRP3, and ASC in FLSs of each group. Compared with the normal group, *p* ^#^ < 0.05; compared with VA + LPS group, *p* ^*^ < 0.05. **(B and C)** Western blot analysis of the effects of VA on procaspase-1, p10, NLRP3, and ASC in rat synovial cells in cell experiments. Compared with the normal group, *p*# < 0.05; compared with VA + LPS group, p* < 0.05. **(D)** Relative gene expression of IL-18 and IL-1β in rat synovial cells in cell experiments. Compared with the normal group, *p*# < 0.05; compared with VA + LPS group, p* < 0.05. Western blot analysis of the effects of VA on IL-18 and IL-1β in rat synovial cells in cell experiments **(C and E)**. Compared with the normal group, *p*# < 0.05; compared with VA + LPS group, p* < 0.05.

To explore the effects of VA on inflammation in FLSs, the expressions of caspase-1, NLRP3, and ASC with or without VA intervention after LPS treatment were analyzed (Figure 5). As shown in Figures 5A,B, the mRNA and protein levels of caspase-1, ASC, and NLRP3 were highly expressed in LPS-induced cells (*p* < 0.05), while VA intervention significantly prevented the upregulation of these factors (*p* < 0.05). Moreover, both the gene and protein expressions of IL-18 and IL-1β (Figures 5C–E) were increased in the LPS group (*p* < 0.05), whereas the expressions of these factors in the VA + LPS group were significantly lower than those in the LPS group (*p* < 0.05).

## Discussion

Studies have shown that VA can improve inflammation and relieve pain, but the mechanisms underlying VA treatment remain unknown. In this study, we demonstrated that VA can inhibit the activation of NLRP3 inflammasomes, which are mainly manifested through the downregulation of caspase-1, ASC, and NLRP3, thereby reducing the inflammatory state of synovitis in KOA. VA also inhibited the expression of proinflammatory factors IL-18 and IL-1β, the downstream substances released after the activation of NLRP3 inflammasomes. In addition, VA decreased both the gene and protein expression of the pain-related factors NGF, TrkA, and CGRP, alleviating pain in the KOA rats. Interestingly, we also observed an inhibitory effect of VA treatment on synovial fibrosis in rats. Immunohistochemical staining of collagen I and Sirius red staining of the synovial tissue both showed less collagen deposition after VA intervention.

KOA is a disabling disease for the elderly worldwide. In recent years, the number of osteoarthritis patients has gradually increased. The main features of KOA include synovial inflammation, cartilage destruction, and osteophyte formation. Synovitis occurs in the early stage of KOA and is associated with joint pain and stiffness in patients. For treatment, nonsteroidal anti-inflammatory drugs (NSAIDs) are usually the first-line medications in clinical guidance. These types of drugs have analgesic, antipyretic, and high-dose anti-inflammatory effects but also have many adverse side-effects, especially in the digestive system (Mobasheri, 2013; Rannou et al., 2016). Intra-articularly injected drugs, such as local anesthetics and tranexamic acid, also show cytotoxicity (Busse et al., 2019). Chinese herbal medicines, including decoctions and herbal extracts, are effective in treating KOA with few adverse effects. Therefore, finding a safe anti-inflammatory and analgesic drug among Chinese herbal medicine seems to be a feasible method. In our previous study, we conducted an active component analysis of Sanse powder, a classic prescription for KOA clinical treatment, and identified dozens of potential effector components, including VA. It has been noted that VA has anti-inflammatory and analgesic effects (Calixto-Campos et al., 2015; Lee et al., 2018). However, how VA affects the inflammation of KOA and its effects on pain are still poorly understood. Therefore, we performed this study to determine the potential mechanisms.

Synovitis is a process characterized by thickening of the synovium (hyperplasia and hypertrophy) and cellular infiltration (macrophages and lymphocytes) (Miao et al., 2010; Jorgensen and Miao, 2015). Oehler et al. (2000) reported that cell layer proliferation is the most common phenomenon among early synovial changes in KOA. The NLRP3 inflammasome is also associated with synovitis. The role of VA on inflammatory and oxidative stress has likewise been reported (Calixto-Campos et al., 2015). Lee et al. (2018) also showed that o-vanillic acid has the potential to treat inflammation by inhibiting macrophages. Huang et al. (2019) confirmed that VA may affect chondrocytes through the mitogen-activated protein kinases (MAPKs) and phosphoinositide 3-kinase (PI3K)/protein kinase B (AKT)/nuclear factor kappa-light-chain-enhancer of activated B cells (NF-κB) pathways. Recently, Ziadlou et al. (2020) indicated that VA could inhibit NF-κB signaling through the attenuation of the nuclear factor of the kappa light polypeptide gene enhancer in B-cell inhibitor alpha (IκBα) phosphorylation. Meanwhile, other studies have shown that IL-1β and IL-18 are key inflammatory mediators in the pathological process of synovitis (Rahmati et al., 2016). IL-1β can activate the TIR superfamily of receptors (Ziadlou et al., 2020). Moreover, VA treatment significantly inhibits IL-1β levels (Calixto-Campos et al., 2015; Ziadlou et al., 2020), which indicates that the TIR superfamily of receptors may be a target for VA and its anti-inflammatory properties. However, few studies have shown the effects of VA on NLRP3 inflammasomes. Recent studies have explained the role of NLRP3 inflammatory bodies in the inflammatory cascade. NLRP3 is a multiprotein oligomer composed of caspase-1. Caspase-1 is a speckle-like protein associated with apoptosis and contains CARD (ASC) and NOD-like receptor protein 3 (NLRP3). After activation, NLRP3 interacts with ASC, which can bridge NLRP3 to procaspase-1, and procaspase-1 can activate caspase-1. Activated caspase-1 cleaves the original forms of IL-1β and IL-18 into mature and active forms (Groslambert and Py, 2018; Krishnan et al., 2014; Lacey et al., 2018). In the present study, VA was shown to interfere with the inflammatory pathway, thereby reducing the expression of caspase-1, ASC, and NLRP3 in synovial cells in the KOA environment simulated by LPS and in the KOA model group. VA also inhibited the increases in the expression of the NLRP3 inflammatory bodies and their downstream substances, IL-1β and IL-18, suggesting that VA inhibited the activation of NLRP3 inflammatory bodies, which is consistent with previous studies on NLRP3 inflammasome-driven inflammation (Martinon et al., 2002; Zhang et al., 2019). In this study, we showed that VA can inhibit the activation of NLRP3 inflammasomes by inhibiting the expression of caspase-1, ASC, and NLRP3 proteins, thereby reducing the inflammatory expression in KOA and exerting anti-inflammatory effects. In addition, it also reduced the expression of IL-18 and IL-1β in the downstream substances of the NLRP3 inflammasome.

Inflammation can cause nerve damage, which may be one of the causes of pain. Studies have shown that NGF, TrkA, and CGRP are closely related to OA pain (Denk et al., 2017; Schou et al., 2017). NGF is widely considered a mediator of chronic pain and is mainly expressed in the synovial fluid, OA osteochondral junction, synovium, and cartilage (Montagnoli et al., 2017; Takano et al., 2017a). Animal and population studies have shown that NGF levels increase under trauma, inflammation, and chronic pain (Collison, 2019). TrkA is a functional receptor for NGF and one of the main targets of the NGF pain signaling pathway (Sousa-Valente et al., 2018). Moreover, an increase in CGRP is closely related to the generation and maintenance of chronic pain, and CGRP's sensory neurons dominate most joint structures, including the synovium, ligaments, and subchondral bone, which contribute to peripheral sensitization and inflammation. Therefore, under OA pain sensitization, the expression of CGRP will also increase accordingly (Walsh et al., 2015; Takano et al., 2017b). At the same time, CGRP can enhance or reduce the effects of other chemicals and regulate the excitability of sensory ends after experiencing harmful stimuli (Grässel and Muschter, 2017). Therefore, we subsequently investigated whether VA inhibition in the activation of NLRP3 inflammatory bodies could affect the above mediators. We found that VA can inhibit the upregulation of NGF, TrkA, and CGRP in FLSs induced by LPS. In animal experiments, we found that oral VA also inhibited the upregulation of these pain mediators in the model rats with KOA induced by MIA. These results indicate that VA can reduce the expression of pain mediators in KOA model rats, thereby alleviating osteoarthritis pain. A previous study also showed that VA can reduce inflammation-related pain by inhibiting neutrophil recruitment, oxidative stress, cytokine production, and NF-κB activation (Calixto-Campos et al., 2015).

However, for technical reasons, our study has several limitations. For example, the specific mechanism of VA role in the pain caused by KOA inflammatory response has not been studied in detail, and no further research has been done on the observed synovial fibrosis. At the same time, only one dose of VA was used in this study. Therefore, we did not observe the long-term effects of VA on synovial inflammation. In addition, an animal model of KOA that cannot account for all related factors of human KOA was used in this study.

In summary, our data show that VA can reduce KOA synovial inflammation and inhibit NLRP3 inflammasome activation. In addition, we also observed that VA reduces pain-related behavior/mediator in knee osteoarthritis *in vivo*. Overall, VA may protect knee joints by inhibiting NLRP3 activation.

## Data Availability

The original contributions presented in the study are included in the article/Supplementary Material; further inquiries can be directed to the corresponding author.
